# Definition of the upper reference limit for thyroglobulin antibodies according to the National Academy of Clinical Biochemistry guidelines: comparison of eleven different automated methods

**DOI:** 10.1007/s13317-017-0096-3

**Published:** 2017-06-19

**Authors:** F. D’Aurizio, P. Metus, A. Ferrari, B. Caruso, R. Castello, D. Villalta, A. Steffan, K. Gaspardo, F. Pesente, N. Bizzaro, E. Tonutti, S. Valverde, C. Cosma, M. Plebani, R. Tozzoli

**Affiliations:** 1 Clinical Pathology Institute, University Hospital, P.le S. Maria della Misericordia, 33100 Udine, Italy; 2 Clinical Pathology Laboratory, ‘S. Maria degli Angeli’ Hospital, Pordenone, Italy; 3 Clinical Pathology Laboratory, University Hospital, Verona, Italy; 4 General Medicine and Endocrinology, University Hospital, Verona, Italy; 5 Allergology and Immunology, ‘S. Maria degli Angeli’ Hospital, Pordenone, Italy; 6 Oncological Clinical Pathology Laboratory, CRO, IRCCS, Aviano, Italy; 7 Clinical Pathology Laboratory, ‘S. Antonio Hospital’, Tolmezzo, Italy; 8 Laboratory of Immunopathology and Allergology, University Hospital, Udine, Italy; 9 Laboratory Medicine, ‘Madonna della Navicella’ Hospital, Chioggia (Ve), Italy; 10 Department of Laboratory Medicine, University Hospital, Padua, Italy

**Keywords:** Autoimmune thyroid disease, Thyroglobulin autoantibodies, Upper reference limit, Immunoassay, Harmonization

## Abstract

**Purpose:**

In the last two decades, thyroglobulin autoantibodies (TgAb) measurement has progressively switched from marker of thyroid autoimmunity to test associated with thyroglobulin (Tg) to verify the presence or absence of TgAb interference in the follow-up of patients with differentiated thyroid cancer. Of note, TgAb measurement is cumbersome: despite standardization against the International Reference Preparation MRC 65/93, several studies demonstrated high inter-method variability and wide variation in limits of detection and in reference intervals. Taking into account the above considerations, the main aim of the present study was the determination of TgAb upper reference limit (URL), according to the National Academy of Clinical Biochemistry guidelines, through the comparison of eleven commercial automated immunoassay platforms.

**Methods:**

The sera of 120 healthy males, selected from a population survey in the province of Verona, Italy, were tested for TgAb concentration using eleven IMA applied on as many automated analyzers: AIA-2000 (AIA) and AIA-CL2400 (CL2), Tosoh Bioscience; Architect (ARC), Abbott Diagnostics; Advia Centaur XP (CEN) and Immulite 2000 XPi (IMM), Siemens Healthineers; Cobas 6000 (COB), Roche Diagnostics; Kryptor (KRY), Thermo Fisher Scientific BRAHMS, Liaison XL (LIA), Diasorin; Lumipulse G (LUM), Fujirebio; Maglumi 2000 Plus (MAG), Snibe and Phadia 250 (PHA), Phadia AB, Thermo Fisher Scientific. All assays were performed according to manufacturers’ instructions in six different laboratories in Friuli-Venezia Giulia and Veneto regions of Italy [Lab 1 (AIA), Lab 2 (CL2), Lab 3 (ARC, COB and LUM), Lab 4 (CEN, IMM, KRY and MAG), Lab 5 (LIA) and Lab 6 (PHA)]. Since TgAb values were not normally distributed, the experimental URL (e-URL) was established at 97.5 percentile according to the non-parametric method.

**Results:**

TgAb e-URLs showed a significant inter-method variability. Considering the same method, e-URL was much lower than that suggested by manufacturers (m-URL), except for ARC and MAG. Correlation and linear regression were unsatisfactory. Consequently, the agreement between methods was poor, with significant bias in Bland–Altman plot.

**Conclusions:**

Despite the efforts for harmonization, TgAb methods cannot be used interchangeably. Therefore, additional effort is required to improve analytical performance taking into consideration approved protocols and guidelines. Moreover, TgAb URL should be used with caution in the management of differentiated thyroid carcinoma patients since the presence and/or the degree of TgAb interference in Tg measurement has not yet been well defined.

## Introduction

Human thyroglobulin (Tg) is a high molecular weight (660 kDa) soluble glycoprotein, typically stored within the follicular colloid of the thyroid, acting as the substrate for thyroid hormones (triiodothyronine, T3 and thyroxine, T4). As Tg is produced and utilized entirely by benign or differentiated malignant thyroid cells, it is considered a good tumor marker for patients with differentiated thyroid carcinoma (DTC) [1, 2] after removal of benign and malignant thyroid tissue by surgery and I^131^ ablation. Over the years, advances in assay technologies have led to important improvements in the analytical performances of Tg immunometric assays (IMAs); above all, the functional sensitivity (FS) of Tg IMAs has greatly improved: from 0.5 to 1.0 μg/L of the first generation IMAs to 0.05–0.10 μg/L of the second generation (2G) IMAs [3].

Nevertheless, the major limitation of 2G IMA testing is interference by serum Tg autoantibodies (TgAb) causing, as a rule, underestimation of Tg results and possibly masking disease recurrence [4–6]: it has been hypothesized that the complex between free Tg and endogenous TgAb prevents free Tg from binding to the capture and/or signal monoclonal antibody reagents and/or alternatively, endogenous TgAb binding to free Tg masks the epitopes recognized by monoclonal antibody reagents [5, 7].

Serum TgAb are reported to be present in about 25–30% of DTC patients depending of the assay used and the cut-off employed to classify samples as positive or negative [1, 7]. They are more frequent in females [8] and they are also present in about 60% of patients with autoimmune thyroid disease (AITD) [9]. On the basis of these considerations, the role of TgAb measurement has evolved from a marker of thyroid autoimmunity [10, 11] to a test associated with Tg to investigate TgAb interference [12]. Consequently, serum TgAb have evolved as a surrogate test for tumor marker replacing Tg determination by IMAs, in cases of analytical interference from TgAb [13, 14].

Of note, the measurement of TgAb could be cumbersome. Analytical limitations of serum TgAb assays have been reported in the context of thyroid autoimmunity diagnosis [9]. Despite standardization against the International Reference Preparation (IRP) MRC 65/93, several studies demonstrated a high variability in the analytical performances of different TgAb IMAs: large variation in limits of detection (LOD), FS, inter-method results, reference intervals with poor concordance between TgAb assays in patients with DTC [15–22]. The difficulty in standardization is in part due to the heterogeneous Tg immunoreactivity: differential splicing of Tg mRNA, various post-translational modifications, and alterations of biosynthesis regulation in thyroid tumor cells lead to exposure or masking of epitopes with resulting differences in Tg immunologic structure [23]. Besides Tg heterogeneity, assay discordance has also been assigned to various specificity of circulating TgAb in patient sera [6]. As a result, different TgAb values are obtained when the same serum is tested with different methods [15–22]. Finally, differences in assay reagents, above all the preparation of the antigen (Tg), definitely contribute to assay variability [1–12].

The manufacturers’ upper reference limit (URL) for TgAb, set up to identify patients with AITD but misleading for evaluation of TgAb interference in Tg assay, is another aspect to consider. Reference intervals are the most widely used tool for the interpretation of clinical laboratory results. The Clinical and Laboratory Standards Institute (CLSI) Expert Panel on Reference Values has provided guidelines for the determination of reliable reference intervals (EP28-A3c) [24]. They recommended the use of the direct method, which implies the enrolment of a healthy population of at least 120 individuals and the determination of 2.5th and 97.5th percentile for the lower reference limit and the URL, respectively. As regards thyroid antibodies (thyroid peroxidase antibodies—TPOAb and TgAb) for AITD diagnosis, the 2003 proposal of the National Academy of Clinical Biochemistry (NACB) recommends the use of a direct method and a reference group composed of 120 men younger than 30 years, biochemically euthyroid [i.e., with serum thyrotropin stimulating hormone (TSH), concentrations between 0.5 and 2.0 mIU/L], and without risk parameters (goiter, family history of AITD, or other autoimmune diseases) [25].

However, the definition of the TgAb URL remains a matter of debate, because of the problems in enrolling the appropriate reference group [25] and in the determination of TgAb cut-off suitable for the identification of assay interference and consequently for the use of TgAb as surrogate marker in the follow-up of DTC [12].

Taking into account the above considerations, the main aim of the present study was the determination of TgAb URL, according to the NACB guidelines, by the use of eleven commercial automated IMA platforms. A further aim of the study was to compare the analytical performances of the methods used, in an attempt to evaluate, whenever possible, their effectiveness in detecting TgAb interference.

## Materials and methods

One hundred and twenty male subjects were selected from a population survey in the province of Verona, Italy, according to the NACB criteria [25]. All of them gave informed consent for their participation in the study. Their sera were tested for TgAb concentration by using eleven IMA methods applied in as many automated analyzers: AIA-2000 (AIA) and AIA-CL2400 (CL2), Tosoh Bioscience; Architect (ARC), Abbott Diagnostics; Advia Centaur XP (CEN) and Immulite 2000 XPi (IMM), Siemens Healthineers; Cobas 6000 (COB), Roche Diagnostics; Kryptor (KRY), Thermo Fisher Scientific BRAHMS, Liaison XL (LIA), Diasorin; Lumipulse G (LUM), Fujirebio; Maglumi 2000 Plus (MAG), Snibe and Phadia 250 (PHA), Phadia AB, Thermo Fisher Scientific. All assays were performed according to manufacturers’ instructions at six different laboratories in Friuli-Venezia Giulia and Veneto regions of Italy [Lab 1 (AIA), Lab 2 (CL2), Lab 3 (ARC, COB and LUM), Lab 4 (CEN, IMM, KRY and MAG), Lab 5 (LIA) and Lab 6 (PHA)]. The main features of the eleven methods are summarized in Table 1. All methods are standardized with the reference preparation (IRP MRC 65/93) and use International Units (IU), except for CEN and KRY whose results were initially expressed in Arbitrary Units and then converted in IU (Table 1). The normality of the distribution was assessed using the Shapiro–Wilk test. Since TgAb values were not normally distributed, the experimental URL (e-URL) was established at 97.5th according to the non-parametric percentile method (CLSI standard C28-A3c) [24]. Moreover, the non-parametric Kruskal–Wallis test and the Dunn’s multiple comparison test were used for comparing the median values of the eleven groups.

**Table 1:** Analytical performance characteristics of the current TgAb automated immunoassays

Method	Immunoassay principle	Tracer/enzyme	Assay type	Imprecision (%):intra-; inter-; total	LoD^d^ (IU/mL)	LoQ^d^ (IU/mL)	Assay range (IU/mL)
AIA	FEIA	4MUP/ Alkaline phosphatase	NC	4.3–5.1; nd; 5.5–6.0	0.12	nd	0.12–2000
ARC	CLIA	Acridinium esters	NC	1.7–6.6^b^; nd; 2.7–8.2^b^	0.07	0.31	0.07–1000
CEN^a^	CLIA	Acridinium esters	C	2.9–5.5; 1.8–2.0; 3.5–5.8	10	30^e^	10–500
CL2	CLEIA	Difurat^®^	NC	5.1–5.5; 5.8–6.6; nd	0.005	nd	0.005–2500
COB	ECLIA	Ruthenium derivatives	C	1.3–5.6^c^; 2.1–8.7^c^; nd	10	nd	10–4000
IMM	CLIA	Adamantyl dioxetane phosphate/Alkaline phosphatase	NC	3.2–4.9; 4.6–5.8; nd	2.2	nd	20–3000
KRY^a^	TRACE	Europium cryptate/ XL 665	C	1.5–3.5; 6.8–20.0; nd	10	33	10–850
LIA	CLIA	Isoluminol derivatives	NC	2.3–3.2; 4.4–8.9; nd	5	10	5–5000
LUM	CLEIA	AMPPD	NC	1.8–4.6; nd; 2.5–5.3^c^	5.152	5.152	5.152–3000
MAG	CLIA	ABEI	NC	2.8–9.1; 5.2–9.8; nd	10	nd	10–2800
PHA	FIA	4-methyl-umbellipheryl-β-d-galactoside/β-galactosidase	NC	3.3–5.6; 2.6–6.5; nd	12	nd	12–4794

*4MUP* 4-methyl-umbelliferyl phosphate, ABEI *N*-(aminobutil)-*N*-(ethyl)-isoluminol, *AIA* AIA-2000, Tosoh Bioscience, *AMPPD* alkaline phosphatase-spiroadamantyl-methoxy-phosphoryloxy-phenyl-dioxetane, *ARC* Architect, Abbott Diagnostics, *C* competitive immunoassay, *CEN* Advia Centaur XP, Siemens Healthineers, *CL2* AIA CL-2400, Tosoh Bioscience, *CLIA* chemiluminescence immunoassay, *CLEIA* chemiluminescence enzyme immunoassay, *COB* Cobas 6000, Roche Diagnostics, *ECLIA* electrochemiluminescence immunoassay, *FEIA* fluorescence enzyme immunoassay, *FIA* fluoroimmunoassay, *IMM* Immulite 2000 XPi, Siemens Healthineers, *KRY* Kryptor, Thermo Fisher Scientific BRAHMS, *LIA* Liaison XL, Diasorin, *LUM* Lumipulse G, Fujirebio, *MAG* Maglumi 2000 Plus, Snibe, *NC* non-competitive immunoassay, *nd* not declared, *PHA* Phadia 250, Phadia AB, Thermo Fisher Scientific, *TRACE* time resolved amplified cryptate emission

^a^All methods are standardized with the reference preparation MRC 65/93 and use International Units (IU/mL) except for Centaur and Kryptor which refer to a secondary standard and use Arbitrary Units (AU/mL); to obtain IU multiply for the conversion factor 2.8 (CEN) and 7.14 (KRY)

^b^Precision defined by the NCCLS Protocol EP5-A [26]

^c^Precision defined by the modified NCCLS Protocol EP5-A2 [27]

^d^LoD and LoQ defined by the CLSI protocol EP17-A [28]

^e^Functional sensitivity defined as TgAb concentration with total CV ≤20%, determined for a period of two days using one lot of reagents and testing, by four instruments, multiple samples from normal patients

The inter-method variability was assessed considering the interquartile range (25th and 75th percentile). To compare the eleven methods, ARC was regarded as the reference assay since it showed a satisfactory combination between the LoD and the assay imprecision (Table 1). Correlation between assays was assessed by Spearman Rank correlation coefficient (*r*_s_); Passing-Bablok regression was applied to verify the linear association between methods, while agreement between assays was analyzed by Bland–Altman plot considering the difference between ARC and the other ten methods (AIA, CEN, CL2, COB, IMM, KRY, LIA, LUM, MAG and PHA). The difference between manufacturer’s URL (m-URL) and e-URL was expressed as the ratio between them in percentage (Delta% = |m-URL − e-URL|/m-URL × 100). A two-sided value of *p* < 0.05 was considered statistically significant. Statistical analyses were performed by GraphPad Prism Software, version 4.0 (San Diego, CA, USA) and MedCalc software, version 11.6 (Ostend, Belgium).

## Results

**Fig. 1:**
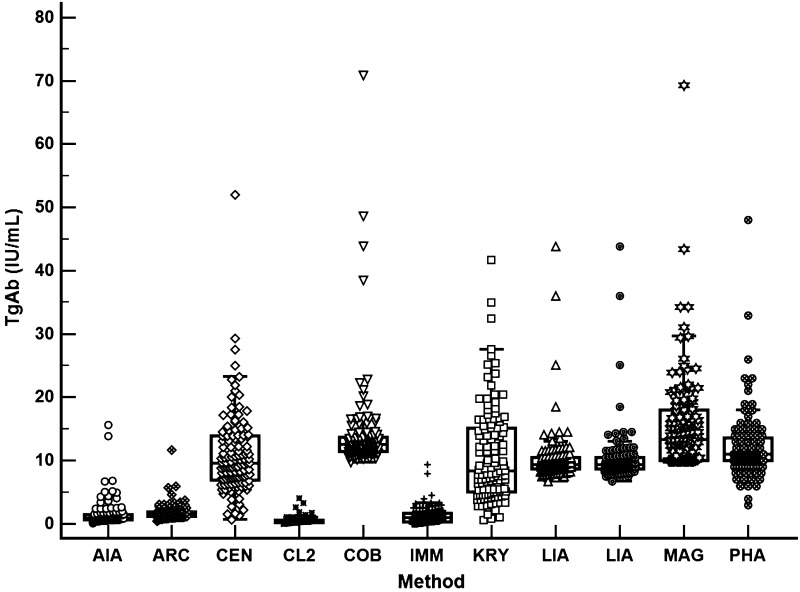
Distribution of TgAb values for each method. *AIA* AIA-2000, Tosoh Bioscience, *ARC* Architect, Abbott Diagnostics, *CEN* Advia Centaur XP, Siemens Healthineers, *CI* confidence intervals, *CL2* AIA CL-2400, Tosoh Bioscience, *COB* Cobas 6000, Roche Diagnostics, *IMM* Immulite 2000 XPi, Siemens Healthineers, *KRY* Kryptor, Thermo Fisher Scientific BRAHMS, *LIA* Liaison XL, Diasorin, *LUM* Lumipulse G, Fujirebio, *MAG* Maglumi 2000 Plus, Snibe, *No.* number, *PHA* Phadia 250, Phadia AB, Thermo Fisher Scientific, *RSD* relative standard deviation. *SD* standard deviation

TgAb results showed a relevant inter-method variability with wide interquartile ranges: the difference reached 48 times for the 25th percentile (minimum: 0.24 IU/mL and maximum: 11.5 IU/mL) and 30 times for 75th percentile (minimum: 0.59 IU/mL, maximum: 17.97 IU/mL) (Fig. 1) (Table 2).

**Table 2:** Summary statistics of TgAb measurements for each method

Method	AIA	ARC	CEN^a^	CL2	COB	IMM	KRY^a^	LIA	LUM	MAG	PHA
No.	120	120	120	120	120	120	120	120	120	120	120
Mean (95% CI)	1.56 1.18–1.95	1.81 1.58–2.03	10.93 9.74–12.13	0.53 0.43–0.63	14.23 12.87–15.58	1.28 1.04–1.53	10.79 9.43–12.14	10.38 9.57–11.2	7.94 7.2–8.69	15.61 14.19–17.03	12.05 11.09–13.01
SD	2.12	1.26	6.61	0.56	7.52	1.36	7.48	4.49	4.12	7.87	5.33
RSD	1.36	0.7	0.6	1.06	0.53	1.05	0.69	0.43	0.52	0.50	0.44
Median (95% CI)	0.91 0.8–1.08	1.51 1.39–1.61	9.61 8.81–10.6	0.4 0.32–0.46	12.56 11.93–12.84	1.03 0.81–1.21	8.41 7.27–10.49	9.4 9.11–9.81	6.87 6.36–7.58	13.35 12.38–15.07	11 10–11
25th–75th *p*	0.63–1.48	1.21–1.92	6.96–13.83	0.24–0.59	11.5–13.61	0.36–1.65	5.04–15.15	8.66–10.42	5.63–8.64	10.05–17.97	10–13.5
2.5th–97.5th *p*	0.36–6.75	0.73–5.14	1.64–26.25	0.1–2.25	10.31–41.15	0.02–4.23	1.22–29.94	7.50–21.79	5.15–20.56	10.05–34.23	6–24.5
Normal distribution	<0.0001	<0.0001	<0.0001	<0.0001	<0.0001	<0.0001	<0.0001	<0.0001	<0.0001	<0.0001	<0.0001

Mean, SD, median, percentiles are expressed in IU/mL

*AIA* AIA-2000, Tosoh Bioscience, *ARC* Architect, Abbott Diagnostics, *CEN* Advia Centaur XP, Siemens Healthineers, *CI* confidence intervals, *CL2* AIA CL-2400, Tosoh Bioscience, *COB* Cobas 6000, Roche Diagnostics, *IMM* Immulite 2000 XPi, Siemens Healthineers, *KRY* Kryptor, Thermo Fisher Scientific BRAHMS, *LIA* Liaison XL, Diasorin, *LUM* Lumipulse G, Fujirebio, *MAG* Maglumi 2000 Plus, Snibe, *No*. number, *p* percentile, *PHA* Phadia 250, Phadia AB, Thermo Fisher Scientific, *RSD* relative standard deviation, SD *standard deviation*

^a^The results obtained with Advia Centaur XP and Kryptor Compact Plus are expressed as arbitrary units (AU/mL); to obtain IU multiply for the conversion factor 2.8 (CEN) and 7.14 (KRY)

A statistically significant difference between medians was observed for all methods except for 11 pairs of the 45 combinations analyzed (Fig. 1) (Table 3).

**Table 3:** Kruskal–Wallis test and Dunn’s multiple comparison test of TgAb methods: comparison of all pairs of columns

A. Kruskal–Wallis test	
No. of groups	11
*p* Value	*p* < 0.0001
Do the medians vary significantly (*p* < 0.05)	Yes

B. Dunn’s multiple comparison test	*p* value
AIA vs ARC	*p* > 0.05
AIA vs IMM	*p* > 0.05
ARC vs IMM	*p* > 0.05
CEN vs KRY	*p* > 0.05
CEN vs LIA	*p* > 0.05
CEN vs LUM	*p* > 0.05
CL2 vs IMM	*p* > 0.05
COB vs MAG	*p* > 0.05
KRY vs LIA	*p* > 0.05
KRY vs LUM	*p* > 0.05
LIA vs LUM	*p* > 0.05

A There is a significant difference between the medians of the eleven groups. B In the table are reported only the comparison of groups with non-significant differences

*AIA* AIA-2000, Tosoh Bioscience, *ARC* Architect, Abbott Diagnostics, *CEN* Advia Centaur XP, Siemens Healthineers, *CI* confidence intervals, *CL2* AIA CL-2400, Tosoh Bioscience, *COB* Cobas 6000, Roche Diagnostics, *IMM* Immulite 2000 XPi, Siemens Healthineers, *KRY* Kryptor, Thermo Fisher Scientific BRAHMS*, LIA* Liaison XL, Diasorin, LUM, Lumipulse G, Fujirebio, *MAG,* Maglumi 2000 Plus, Snibe, *No.* number, *PHA* Phadia 250, Phadia AB, Thermo Fisher Scientific

e-URLs differed from one method to the other. Of note, within the same method, e-URL was much lower than m-URL, except for ARC and MAG, which showed similar values for both (Table 4).

**Table 4:** Experimental upper reference limit compared to the manufacturer’s upper reference limit for most of the current TgAb automated immunoassays, established from a cohort of 120 euthyroid control subjects

Method	No.	m-URL (IU/mL)	e-URL (C.I. 90%) (IU/mL)	Delta (%)
AIA	120	13.6	6.82 (5–15.7)	49.85
ARC	120	4.11	5.66 (3.29–11.64)	37.71
CEN^a^	120	60	27.44 (21.9–52)	54.27
CL2	120	6.8	2.63 (1.15–4.08)	61.32
COB	120	115	43.69 (21.16–70.88)	62
IMM	120	40	4.46 (3.26–9.41)	88.85
KRY^a^	120	33	32.23 (25.14–41.61)	2.33
LIA	120	100	24.93 (14.37–43.91)	75.07
LUM	120	55.4	21.07 (12.3–32.8)	61.97
MAG	120	30	34.23 (29.46–69.36)	14.1
PHA	120	60	25.93 (21–48)	56.78

e-URL: 97.5th percentile; Delta = |m-URL − e-URL|/m-URL × 100

*AIA* AIA-2000, Tosoh Bioscience, *ARC* Architect, Abbott Diagnostics, *CEN* Advia Centaur XP, Siemens Healthineers*, CI* confidence intervals, *CL2* AIA CL-2400, Tosoh Bioscience, *COB* Cobas 6000, Roche Diagnostics, *e*-*URL* experimental upper reference limit, *IMM* Immulite 2000 XPi, Siemens Healthineers, *KRY* Kryptor, Thermo Fisher Scientific BRAHMS, *LIA* Liaison XL, Diasorin, *LUM* Lumipulse G, Fujirebio, *m*-*URL* manufacturer upper reference limit, *MAG* Maglumi 2000 Plus, Snibe, *No.* number, *PHA* Phadia 250, Phadia AB, Thermo Fisher Scientific

^a^The results obtained with Advia Centaur XP and Kryptor Compact Plus are expressed as arbitrary units (AU/mL); to obtain IU multiply for the conversion factor 2.8 (CEN) and 7.14 (KRY)

**Fig. 2:**
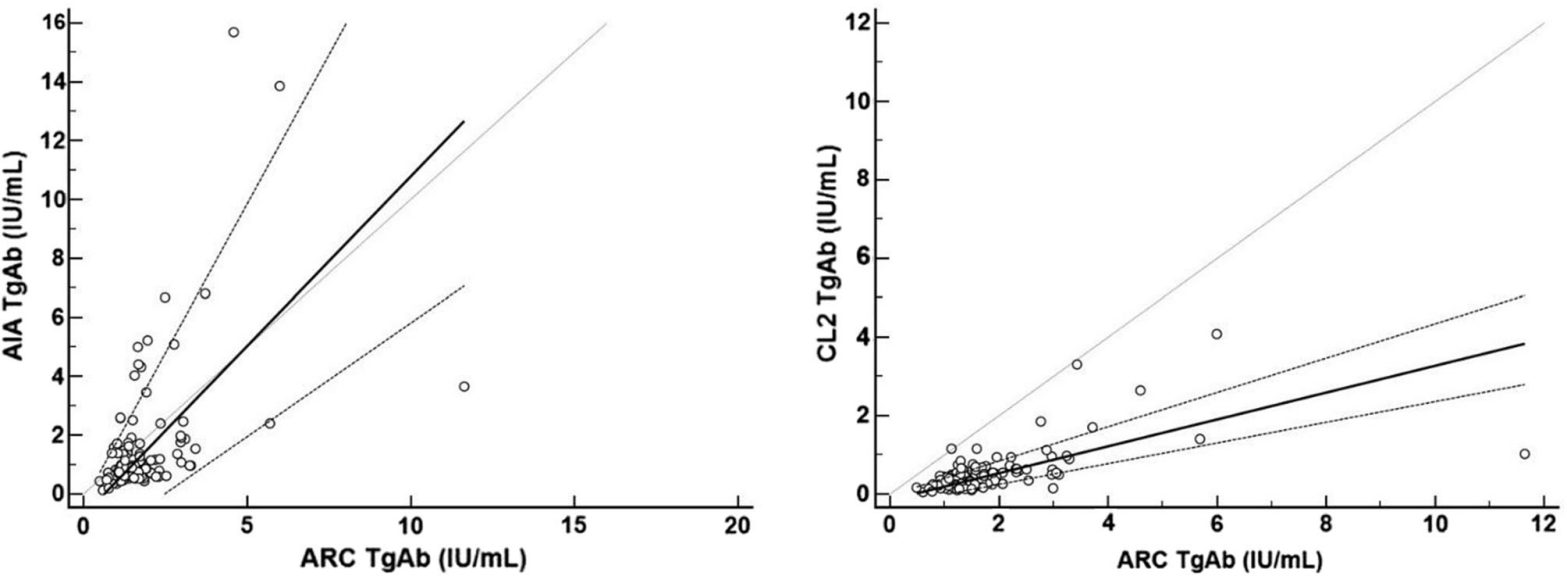
Passing-Bablok regression of TgAb methods. ARC was chosen as the reference method on the *x* axis. ARC vs AIA and ARC vs CL2 showed the best relationship in terms of slope and intercept. *AIA* AIA-2000, Tosoh Bioscience, *ARC* Architect, Abbott Diagnostics, *CL2* AIA CL-2400, Tosoh Bioscience

**Fig. 3:**
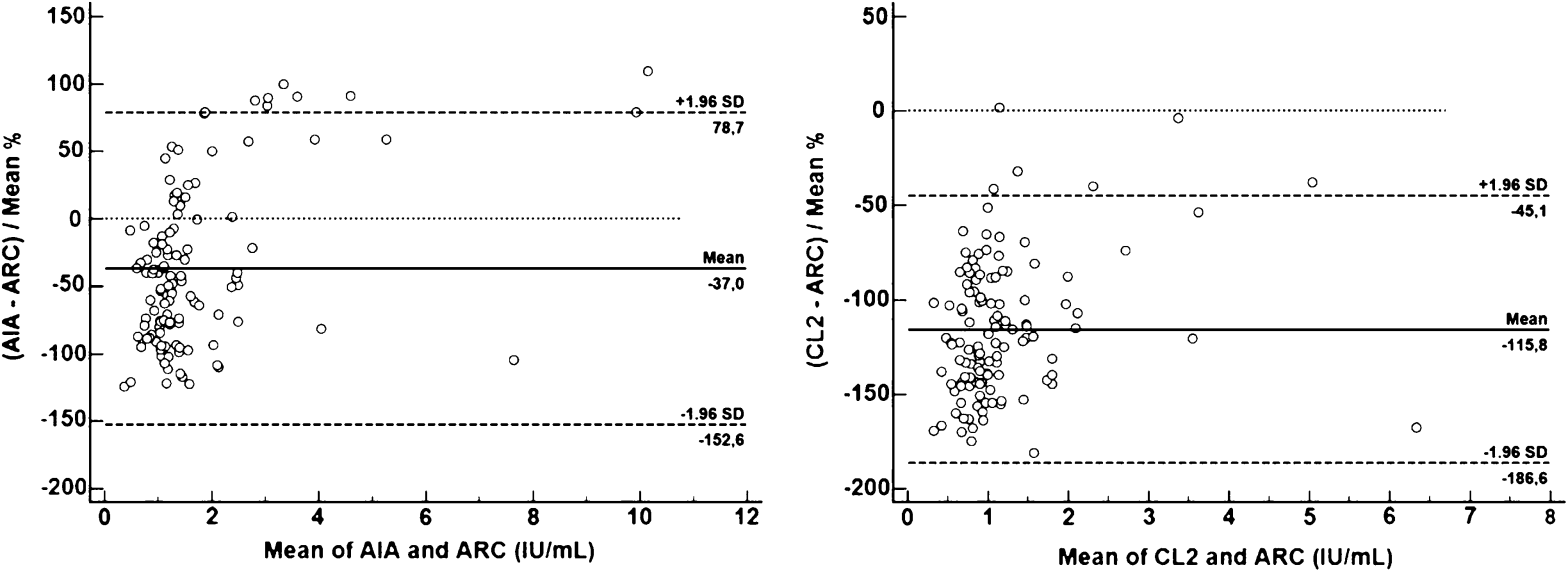
Bland-Altman plots showing the difference between ARC and AIA and between ARC and CL2. ARC was chosen as the reference method. An ideal mean difference of 0 is indicated by a dotted line, the mean difference by a *solid line* and the limits of agreement for the mean difference, as defined by 95% confidence limits, by *dashed lines*. *AIA* AIA-2000, Tosoh Bioscience, *ARC* Architect, Abbott Diagnostics, *CL2* AIA CL-2400, Tosoh Bioscience

As regards the correlations between methods, *r*_s_ ranged from 0.17 (ARC vs CEN) to 0.56 (ARC vs CL2) (Table 5). Using Passing-Bablok analysis, TgAb method comparison resulted in varying degrees of agreement with the reference method (ARC). Slopes were all far from 1 except for ARC vs AIA (slope = 1.15) and ARC vs CL2 (0.34) (Fig. 2) (Table 5); intercepts varied from −29.92 to 3.7, they were far from 0 except for ARC vs AIA (−0.75) and ARC vs CL2 (−0.15) (Fig. 2) (Table 5). Subsequently, a relevant positive or negative mean biases were observed by Bland–Altman analysis ranging from −115.8% (CL2 vs ARC) to 156.4% (MAG vs ARC). The best agreement was between AIA and ARC with a mean bias of −37% (Fig. 3) (Table 6).

**Table 5:** Summary of method comparison by Passing-Bablok regression and Spearman’s rank correlation for the TgAb methods

Comparison	ARC vs AIA	ARC vs CEN	ARC vs CL2	ARC vs COB	ARC vs IMM	ARC vs KRY	ARC vs LIA	ARC vs LUM	ARC vs MAG	ARC vs PHA
No.	120	120	120	120	120	120	120	120	120	120
Slope (95% CI)	1.15 (0.77–2.02)	26.56 (16.24–63.2)	0.34 (0.26–0.44)	5.98 (0.32 – 8.12)	1.92 (1.42–2.64)	38.36 (21.22–94.83)	5.45 (3.48–7.52)	7.03 (5.27–10.57)	17.99 (11.86–32.26)	8.33 (5.77–13.33)
*y*-Intercept (95% CI)	−0.75 (−1.92 to −0.26)	−29.92 (−85.36 to −14.53)	−0.15 (−0.29 to −0.03)	3.7 (0.67–6.39)	−1.9 (−2.96 to −1.17)	−47.31 (−132.72 to −22.98)	1.51 (−1.58 to 4.22)	3.51 (−8.71 to −0.77)	−13.14 (−33.37 to −4.24)	−1.63 (−9.07 to 2.15)
Equation	*y* = −0.75 + 1.15*x*	*y* = −29.92 + 26.56*x*	*y* = −0.15 + 0.34*x*	*y* = 3.7 + 5.98*x*	*y* = −1.9 + 1.92x	*y* = −47.31 + 38.36*x*	*y* = 1.51 + 5.45*x*	*y* = −3.51 + 7.03*x*	*y* = −13.14 + 17.99*x*	y = −1.63 + 8.33*x*
*r*_s_ (95% CI)	0.41 (0.24–0.55)	0.17 (−0.01–0.34)	0.56 (0.42–0.67)	0.49 (0.34–0.61)	0.28 (0.1–0.43)	0.19 (0.01–0.35)	0.28 (0.11–0.44)	0.18 (−0.01–0.35)	0.23 (0.05–0.39)	0.35 (0.18–0.49)

The *y*-intercept is expressed as IU/mL

*AIA,* AIA-2000, Tosoh Bioscience, *ARC* Architect, Abbott Diagnostics, *CEN* Advia Centaur XP, Siemens Healthineers, *CI* confidence intervals, *CL2* AIA CL-2400, Tosoh Bioscience, *COB* Cobas 6000, Roche Diagnostics*, e*-*URL* experimental upper reference limit, *IMM* Immulite 2000 XPi, Siemens Healthineers, *KRY* Kryptor, Thermo Fisher Scientific BRAHMS, *LIA* Liaison XL, Diasorin, *LUM* Lumipulse G, Fujirebio, *m*-*URL* manufacturer upper reference limit, *MAG* Maglumi 2000 Plus, Snibe, *No.* number, *PHA* Phadia 250, Phadia AB, Thermo Fisher Scientific, *r*_*s*_ Spearman’s rank correlation coefficient

**Table 6:** Summary of method agreement (Bland–Altman plot) for the TgAb methods

Agreement	AIA vs ARC	CEN vs ARC	CL2 vs ARC	COB vs ARC	IMM vs ARC	KRY vs ARC	LIA vs ARC	LUM vs ARC	MAG VS ARC	PHA vs ARC
No.	120	120	120	120	120	120	120	120	120	120
Bias % (±1.96 SD)	−37 (78.7 to −152.6)	133.6 (219.2 – 48)	−115.8 (−45.1 to −186.6)	155.1 (194.3–115.9)	−54.6 (106.8–216.1)	125.8 (226.7–24.8)	141.8 (189.5–94)	125 (188.3–61.7)	156.4 (201.7–111)	147.2 (190.1–104.2)

*AIA* AIA-2000, Tosoh Bioscience, *ARC* Architect, Abbott Diagnostics, *CEN* Advia Centaur XP, Siemens Healthineers*, CI* confidence intervals, *CL2* AIA CL-2400, Tosoh Bioscience, *COB* Cobas 6000, Roche Diagnostics, *e*-*URL* experimental upper reference limit, *IMM* Immulite 2000 XPi, Siemens Healthineers, *KRY* Kryptor, Thermo Fisher Scientific BRAHMS, *LIA* Liaison XL, Diasorin, LUM Lumipulse G, Fujirebio, *m*-*URL* manufacturer upper reference limit, *MAG* Maglumi 2000 Plus, Snibe, *No.* number, PHA Phadia 250, Phadia AB, Thermo Fisher Scientific, *r*_s_ Spearman’s rank correlation coefficient

## Discussion

The determination of the cut-off for the definition of TgAb positivity is an important and controversial issue.

In this study, we have determined the TgAb URL in a reference group of male individuals, meticulously defined as being free of thyroid diseases, by eleven IMA methods, currently used in autoimmunology laboratories, and compared to each other. Actually, to our knowledge, no similar data are present in literature: in the past, other studies faced the same topic but with small numbers of different analytical methods, most of which are no longer in use [9, 15–22, 29].

The first relevant result of the present study was the demonstration of differences between TgAb URLs claimed in the package insert (m-URL) and those obtained in the male reference sample (e-URL): with the exception of ARC and MAG method, e-URLs were lower than those proposed by the manufacturers, the difference ranging from 2.33 to 88.85%. These results were similar to those described in two previous studies dealing with the definition of TPOAb reference limits, determined by several current IMA platforms [30, 31]. In our opinion, these discrepancies could be related to the lack of strict criteria in the selection of the subjects for the reference group. Specifically, racial differences could play some role, as most of the studies, sponsored by manufacturers, were performed in the geographical area of the production line and consequently difficult to reproduce in other settings. Moreover, the use of non-stringent criteria in the choice of subjects could have led to the enrolment of individuals with subclinical AITD, thus resulting in relatively high levels of TgAb causing the raise of the 97.5th percentile of the reference value distribution platforms [32–37].

The second relevant consideration that emerged from the present study was the variation of e-URLs according to the method used. The e-URL ranged from 2.25 (CL2) to 41.15 IU/mL (COB), with an approximately 18-fold variation, consistent with a previous paper which reported the same magnitude of variation using five IMA methods distinct from those considered in the present study (18). The difference between e-URLs supports concerns regarding inter-method variation [38]. Specifically, there were relevant differences between methods in terms of medians (31-fold) (*p* < 0.05, Kruskal–Wallis test) and interquartile ranges. These discrepancies were not expected and not easily explained; in fact, in recent decades, there have been significant improvements in harmonization between methods [39], resulting from the high level of automation of analytical procedures and the use of the same reference preparation (IRP MRC 65/93). Moreover, analytical imprecision seems not contribute to the above differences, as the values declared by the individual manufacturer were essentially overlapping (although obtained with different protocols, some of them standardized, some others not) and in general lower than 10% for both intra- and inter-assay imprecision (Table 1). Such discordance between TgAb assays could be attributed to various factors, including: (1) TgAb heterogeneity which is often independent to standardization efforts, and which implies different specificity for Tg antigen; (2) Tg interference and (3) differences in assay reagents, including solid phase material and the preparations of the antigen (Tg), which could affect the proper exposure of the immunodominant epitopes. Another important aspect to consider, to explain inter-method variability, was the diverse assay structures of the eleven IMA methods leading to a different LoD (Table 1) ranging from 0.005 to 12 IU/mL. Especially, a clear-cut discrepancy between methods with a LoD lower than 0.2 IU/mL (ARC, AIA and CL2) and methods with a LoD equal to or higher than 2 IU/mL was apparent.

To better evaluate the relationship between methods, ARC was chosen as the reference method on the basis of the best combination between LoD and imprecision (Table 1): the correlation of ARC with the other methods was not satisfactory, in line with the variability of the results, broadly described above. Passing-Bablok regression did not show a satisfactory agreement between assays. Furthermore, consistent with regression results, Bland–Altman plot highlighted a statistically significant positive or negative mean biases.

The lack of acceptable agreement between methods has relevant practical implications: clinicians have to use the same method to monitor TgAb concentration in the follow-up of DTC, on the other hand, laboratories must keep users timely informed about any modification in TgAb method to simplify re-baselining.

Despite the analysis of the data showed satisfactory analytical performances of some methods in terms of LoD, being able to measure also low levels of TgAb with adequate precision, the main limitation to this study lay in having contributed only indirectly to the debated question of TgAb analytical interference. In fact, the obtained results did not prove but only suggested the opportunity to choose the more sensitive and accurate latest generation methods for measuring TgAb, to better detect any false negative results even in patients with TgAb levels lower than the cut-off (the so-called “negative patient”). Therefore, according to these considerations, two different cut-offs for TgAb could be proposed, one for the diagnosis of AITD and one for the effects of TgAb on Tg measurement.

## Conclusions

In spite of the attempt of harmonization, quantitative agreement between methods was generally not satisfactory and methods could not be used interchangeably. Therefore, additional standardization efforts are required to improve analytical performance, and biomedical industries are strongly invited to re-evaluate their assays taking into consideration CLSI approved protocols and guidelines.

Finally, as long as the relationship between TgAb concentration and interference in Tg measurement is not clearly defined, TgAb URL must be used with caution, taking into account that it is usually set for the diagnosis of AITD and not for the identification of potential interference in Tg assay.

## Abbreviations

2G: Second generation
AIA: AIA-2000, Tosoh Bioscience
ARC: Architect, Abbott Diagnostics
CEN: Advia Centaur XP, Siemens Healthineers
CL2: AIA-CL2400 (CL2), Tosoh Bioscience
CLSI: Clinical and Laboratory Standards Institute
COB: Cobas 6000, Roche Diagnostics
DTC: Differentiated thyroid carcinoma
FS: Functional sensitivity
IMA: Immunometric assay
IMM: Immulite 2000 XPi, Siemens Healthineers
KRY: Kryptor, Thermo Fisher Scientific BRAHMS
LIA: Liaison XL, Diasorin
LOD: Limit of detection
LUM: Lumipulse G, Fujirebio
MAG: Maglumi 2000 Plus, Snibe
NACB: National Academy of Clinical Biochemistry
PHA: Phadia 250, Phadia AB, Thermo Fisher Scientific
Tg: Thyroglobulin
TgAb: Thyroglobulin autoantibodies
TPOAb: Thyroid peroxidase antibodies
URL: Upper reference limit
